# Hidden and apparent twins in uranyl-oxide minerals agrinierite and rameauite: a demonstration of metric and reticular merohedry

**DOI:** 10.1107/S1600576721009663

**Published:** 2021-11-02

**Authors:** Jakub Plášil, Václav Petříček, Radek Škoda, Nicolas Meisser, Anatoly V. Kasatkin

**Affiliations:** a Institute of Physics ASCR, v.v.i., Na Slovance 2, Prague 8, 18221, Czech Republic; b Department of Geological Sciences, Masaryk University, Kotlářská 2, Brno, 61137, Czech Republic; c Musée cantonal de géologie, Université de Lausanne, Anthropole, Dorigny CH-1015, Switzerland; d Fersman Mineralogical Museum of the Russian Academy of Sciences, Leninsky Prospekt 18-2, Moscow 119071, Russian Federation

**Keywords:** agrinierite, rameauite, twinning, merohedry, *Jana2020*

## Abstract

In this work, the structures of chemically related uranyl-oxide minerals agrinierite and rameauite have been revisited and some corrections to the available structure data are provided. Both structures were found to be twinned. The two minerals are chemically similar, and though their structures differ considerably, their unit-cell metrics are similar. Agrinierite was found to be twinned by metric merohedry (diffraction type I), whereas the structure of rameauite is twinned by reticular merohedry (diffraction type II). The twinning of the monoclinic unit cells (true cells) leads to pseudo-orthorhombic or pseudo-tetragonal supercells in the single-crystal diffraction patterns of both minerals. According to the new data and refinement, agrinierite is monoclinic (space group *Cm*), with *a* = 14.069 (3), *b* = 14.220 (3), *c* = 13.967 (3) Å, β = 120.24 (12)° and *V* = 2414.2 (12) Å^3^ (*Z* = 2). The twinning can be expressed as a mirror in (101) (apart from the inversion twin), which leads to a supercell with *a* = 14.121, *b* = 14.276, *c* = 24.221 Å and *V* = 2 × 2441 Å^3^, which is *F* centered. The new structure refinement converged to *R* = 3.54% for 6545 unique observed reflections with *I* > 3σ(*I*) and GOF = 1.07. Rameauite is also monoclinic (space group *Cc*), with *a* = 13.947 (3), *b* = 14.300 (3), *c* = 13.888 (3) Å, β = 118.50 (3)° and *V* = 2434.3 (11) Å^3^ (*Z* = 2). The twinning can be expressed as a mirror in (101) (apart from the inversion twin), which leads to a supercell with *a* = 14.223, *b* = 14.300, *c* = 23.921 Å and *V* = 2 × 2434 Å^3^, which is *C* centered. The new structure refinement of rameauite converged to *R* = 4.23% for 2344 unique observed reflections with *I* > 3σ(*I*) and GOF = 1.48. The current investigation documented how peculiar twinning can be, not only for this group of minerals, and how care must be taken in handling the data biased by twinning.

## Abstract 2

Two examples of twinning, by metric and reticular merohedry, in uranyl-oxide minerals demonstrate the care that must be taken during structural studies, and not only of such complex materials. This contribution also demonstrates the possibilities of the *Jana2020* program in revealing twinning and in subsequent refinement.

## 1. Introduction

Uranyl-oxide hy­droxy-hydrates (UOHs) represent a fascinating group of minerals and synthetic phases, closely connected with the hydration–oxidation of uranium dioxide, UO_2+*x*_, as nuclear fuel or uraninite (Finch & Ewing, 1992 ▸; Wronkiewicz *et al.*, 1996 ▸; Janeczek *et al.* 1996 ▸; Plášil, 2014 ▸, 2018*a* ▸). They occur in nature as minerals and are among the first alteration products that form during weathering of uraninite (pitchblende) in oxidized zones of U deposits worldwide (Finch, Suksi *et al.*, 1996 ▸; Finch, Cooper *et al.*, 1996 ▸; Plášil, 2018*a* ▸). Numerous studies focused on their structures, solubilities and thermodynamic stabilities were undertaken in the 1990s and at the beginning of the millennium (*e.g.* Casas *et al.*, 1997 ▸; Finch & Murakami, 1999 ▸; Kubatko *et al.* 2006 ▸; Klingensmith *et al.*, 2007 ▸; Gorman-Lewis *et al.*, 2008 ▸) due to the general importance of UOHs in nuclear waste disposal and the environmental chemistry of uranium. Nevertheless, the results of recent scientific efforts have proven even greater complexity and variability of the entire group (Kirkegaard *et al.*, 2019 ▸; Lu, Zhang, Wei *et al.*, 2020 ▸; Lu, Zhang, Aughterson & Zheng, 2020 ▸; Olds *et al.*, 2017 ▸, 2018 ▸; Plášil, 2017 ▸, 2018*b* ▸; Plášil *et al.*, 2018 ▸, 2020 ▸; Zhang *et al.*, 2016 ▸, 2018 ▸, 2019 ▸).

Agrinierite and rameauite are interesting UOHs, having the same type locality, the Margnac mine (Compreignac, Haute-Vienne, Nouvelle-Aquitaine) in France (Cesbron *et al.*, 1972 ▸). Agrinierite, K_2_(Ca_0.65_Sr_0.35_)[(UO_2_)_3_O_3_(OH)_2_]_2_·5H_2_O (Cahill & Burns, 2000 ▸), has been so far the only naturally occurring UOH to contain an essential amount of Sr, reported by Cesbron *et al.* (1972 ▸). This is of interest due to the possible incorporation of ^90^Sr into the alteration phases of spent nuclear fuel. The rameauite structure has been revealed relatively recently by Plášil *et al.* (2016 ▸). The ideal chemical formula of rameauite is K_2_Ca[(UO_2_)_3_O_3_(OH)_2_]_2_·6H_2_O.

According to the structure studies by Cahill & Burns (2000 ▸), agrinierite is orthorhombic (space group *F*2*mm*, *Z* = 16) with *a* = 14.094 (2), *b* = 14.127 (2), *c* = 24.106 (4) Å and *V* = 4799.6 (1) Å^3^. Rameauite is reported (Plášil *et al.*, 2016 ▸) to be monoclinic (space group *Cc*, *Z* = 4) with *a* = 13.9458 (19), *b* = 14.3105 (19), *c* = 13.8959 (18) Å, β = 118.477 (14)° and *V* = 2437.7 (6) Å^3^. The unit-cell volume of rameauite is approximately half that of agrinierite. The structure of agrinierite has been refined to *R* = 6.55% for 2710 unique observed reflections with *I* > 4σ(*I*) and GOF = 0.851 [*SHELXTL* (Sheldrick, 2008 ▸) software used]. The rameauite structure has been refined to *R* = 6.00% for 1696 unique observed reflections with *I* > 3σ(*I*) and GOF = 1.62 [*Jana2006* (Petříček *et al.*, 2014 ▸) software used].

**Figure 1:**
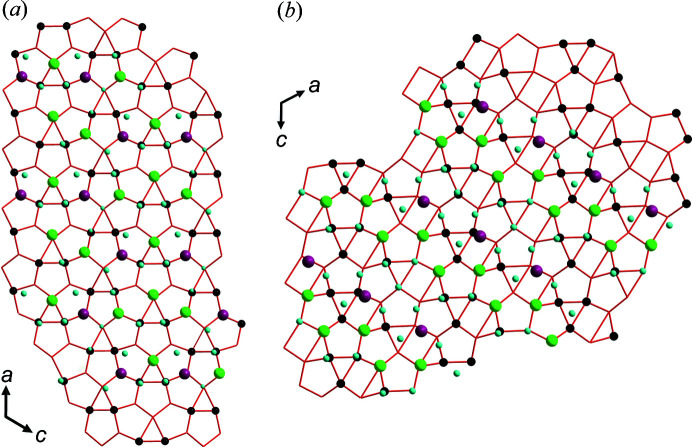
Topology of the UOH layers (red lines) and the distribution of interlayer constituents in (*a*) agrinierite and (*b*) rameauite. The agrinierite UOH sheet is based on the α-U_3_O_8_ type with rods of pentagons and rods of pentagons linked with triangles (oriented up and down). The sheet in rameauite is based on the β-U_3_O_8_ type with rods of pentagons linked with triangles (oriented up and down) and rectangles. Color scheme: Ca/Sr sites are pink, K sites are green, blue spheres are O of molecular H_2_O, black dots within the sheets are OH groups.

While the UOH sheets in the structure of agrinierite are based on the α-U_3_O_8_ type, the sheets in rameauite, despite the overall chemical similarity, are based upon the β-U_3_O_8_ type (Fig. 1 ▸)

The close chemical and structural similarities of the two UOHs prompted us to reinvestigate their structures. This revision led us to the conclusion that both structures are affected by twinning. Here we report the investigation of twinning in both minerals and provide a correct description of the agrinierite unit cell and improved structure models.

## 2. Methodology

### 2.1. Samples studied

The studied specimen of agrinierite originates from the type locality: the former Margnac U mine located about 3 km from Compreignac, Haute-Vienne, Nouvelle-Aquitaine, France (Cesbron *et al.*, 1972 ▸). The studied sample (8 × 4 × 3 mm) is constituted of earthy and massive yellow or orange ‘gummite’ crosscut by inframillimetre-sized veins covered with well shaped orange UOHs, including agrinierite and acicular uranophane-α crystals. In these veins, agrinierite occurs as small (up to 0.8 mm long) pumpkin-orange tabular crystals on {001} pseudo-hexagonal crystals. The sample is preserved in the collection of the Geological Museum of Lausanne, Switzerland (catalog No. MGL 093238). From the same centimetre-sized mineral association, other UOH minerals analyzed utilizing powder X-ray diffraction and energy-dispersive X-ray spectroscopy reflect the distribution of alkaline and alkaline-earth elements on a millimetre scale: compreignacite (K), becquerelite (Ca) and billietite (Ba) (samples MGL 094375–094378).

Rameauite has been studied by Plášil *et al.* (2016 ▸) using the specimen originating from Margnac, France.

### 2.2. Chemical composition of agrinierite

Even though agrinierite was discovered and described *ca* 50 years ago (Cesbron *et al.*, 1972 ▸), its chemical composition remains poorly studied. In the original description, Cesbron *et al.* (1972 ▸) provided only one wet-chemical analysis and, for instance, reported a 2.05 wt% of SrO [∼0.40 Sr atoms per formula unit (apfu)]. Cahill & Burns (2000 ▸) did not provide any chemical data and only gave the composition from the refined structure. The official International Mineralogical Association list of minerals reports agrinierite as K_2_Ca[(UO_2_)_3_O_3_(OH)_2_]_2_·5H_2_O, thus totally neglecting the Sr content in the mineral. In response, we decided to undertake new reliable quantitative chemistry determination by electron microprobe. Crystals of agrinierite were mounted on ep­oxy resin, polished and carbon-coated to determine their chemical compositions utilizing a CAMECA SX100 electron micro­probe. The measurement was performed in wavelength-dispersive mode at 15 kV accelerating voltage, 2 nA beam current and 15 µm beam diameter using the following standards: synthetic UO_2_ for U, synthetic SrSO_4_ for Sr, wollastonite for Ca and sanidine for K. No other elements were above the detection limit. Regardless of the mild analytical conditions, a systematic decrease of K *K*α X-ray intensity during the analysis was observed. Thus, K was analyzed at the beginning of each measurement; the integration time of K was divided into four periods and the concentration was calculated from the values of K *K*α intensities extrapolated to time zero. The raw intensities were processed for matrix corrections using *X-PHI* matrix corrections (Merlet, 1994 ▸) involving a stoichiometric amount of H_2_O. The empirical formula was calculated on the basis of 6 U and the amounts of O, OH and H_2_O were derived from the structure and the rule of electroneutrality. Atomic proportions are shown in apfu (atoms per formula unit).

The agrinierite studied is chemically heterogeneous, particularly in the Ca (0.30–0.61 apfu; 0.83–1.69 wt% CaO) and Sr (0.48–0.67 apfu; 2.49–3.50 wt% SrO) contents. The K content is 1.23–1.38 apfu (2.87–3.23 wt% K_2_O). Considering the average amounts of UO_3_ (85.62 wt%) and H_2_O (6.29 wt%), the average analytical sum is 99.16 wt%. The mean (*n* = 10) empirical formula of agrinierite is K_1.31_(Sr_0.56_Ca_0.47_)[(UO_2_)_3_O_2.84_(OH)_2_]_2_·5H_2_O.

### 2.3. Single-crystal X-ray diffraction

Using single-crystal X-ray diffraction we studied a fragment of the tabular crystal of agrinierite from the Margnac deposit. The crystal of 0.122 × 0.072 × 0.020 mm dimensions was examined at room temperature using a Rigaku SuperNova single-crystal diffractometer. The diffraction experiment was carried out using Mo *K*α radiation (λ = 0.71073 Å) from a micro-focus X-ray tube, collimated and monochromated by mirror optics and detected by an Atlas S2 CCD detector using binning of 2 × 2 pixels and a high-gain mode to register even very weak reflections with an acceptable resolution.

Rameauite was studied using the same instrument; details can be found in the paper by Plášil *et al.* (2016 ▸). Here, we re­analyzed the diffraction data and conducted a new crystal structure refinement. The experimental and refinement details are reported in Table 1 ▸.

## 3. Results

### 3.1. Twinning

The diffraction experiment revealed an *F*-centered ortho­rhombic unit cell similar to that reported by Cahill & Burns (2000 ▸). The initial refinements, using their structure model, led only to a fit with *R* ≃ 8% and a GOF > 2.5. The indexing procedure and unit-cell search in *CrysAlis* (Rigaku, 2019 ▸) did not return satisfactory unambiguous solutions. Therefore we undertook an indexing procedure implemented in *Jana2006* (Tools → GrIndex, using the .tabbin reflection file from *peakhunt*; Petříček *et al.*, 2014 ▸). This procedure revealed a monoclinic unit cell with *a* = 10.0400 (3), *b* = 24.2211 (8), *c* = 10.4020 (3) Å, β = 90.628 (3)° and *V* ≃ 2411.42 Å^3^, indexing about 90.4% of 21 813 reflections. The monoclinic angle close to 90° provides a warning of the possibility of metric merohedral twinning. The test quickly revealed the possibility of twin presence (represented by the mirror in [101]), leading to a supercell with *a* = 14.121, *b* = 14.276, *c* = 24.221 Å and *V* = 2 × 2441.42 Å^3^, which is *F* centered. The monoclinic *I*-centered cell, *a* = 13.9653 (2), *b* = 14.22243 (16), *c* = 13.9675 (2) Å, β = 119.515 (2)° (selected to be similar to that of rameauite) was later transformed during the space-group test procedure in *Jana2006* to *C*2/*m* (14.0694 14.2203 13.9669 90 120.237 90; transformation matrix |1 0 1|0 1 0|1 0 0|). By averaging in *C*2/*m* we obtained 3501 reflections with an *R*_int_ of 5.12% (redundancy of 6.59). The structure solution in *SHELXT* (Sheldrick, 2015 ▸) returned a single solution in *Cm* (Flack 0.24 by *SHELXT* output), which we subsequently refined, with twinning by metric merohedry along with an inversion twin (this involves three symmetry elements to describe the twinning properly). Nevertheless, the refinement was not straightforward. The difficulties were mainly caused by the K sites; for instance, only two (K1 and K4) of the four independent K sites could be refined using a harmonic approach to the atomic displacement parameters. Only one of the K sites allowed the use of an anisotropic description for the atomic displacement parameters, and one of the K sites was found to have a lower occupancy than unity (K2; Table 2 ▸). Nevertheless, the refined occupancies and atomic displacement parameters are probably still greatly affected by the complete overlap of reflections as a result of twinning. For the final cycle of the refinement, the *xyz* coordinates of the U1 atom were fixed due to correlations. The final refinement (Table 1 ▸) for the agrinierite twinned crystal converged to *R* = 3.54% for 6545 unique observed reflections, with *I* > 3σ(*I*) and GOF = 1.07. The final atomic coordinates and displacement parameters for agrinierite are provided in Table 2 ▸, selected interatomic distances in Table 3 ▸ and a bond-valence analysis in Table 4 ▸. The bond-valence analysis was carried out following the procedure by Brown (2002 ▸, 2009 ▸) using bond-valence parameters provided by Gagné & Hawthorne (2015 ▸).

Subsequently, the structure of rameauite was reinvestigated and tested for twinning presence, following the same procedure as for agrinierite. We used the same reflection file as used in the study by Plášil *et al.* (2016 ▸), but we reprocessed it with a newer version of the *CrysAlis* software (version 40.64.67a). We employed a new structure solution and refinement using this reflection file for consistency [we emphasize that by using the original reflection file from the study by Plášil *et al.* (2016 ▸) and a twin-handling procedure in the current version of the *Jana* software (*Jana2020*), several problems occur, which can simply be overcome by using newly processed reflection files from the original diffraction frames]. The unit cell of rameauite, *a* = 13.947 (3), *b* = 14.300 (3), *c* = 13.888 (3) Å, β = 118.50 (3)° with *V* = 2434.3 (11) Å^3^, aligns with previous work (Plášil *et al.*, 2016 ▸). The structure was solved using *SHELXT* in the monoclinic space group *Cc* (Flack 0.42 by *SHELXT* output). The structure refinement involved an inversion twin due to merohedry and, in the final stages, also a reticular twin contribution, finally featuring eight twin elements (due to the group > subgroup relationship between tetragonal > monoclinic symmetry groups). As some of the twin-domain fractions returned slightly negative values they were fixed to 0; the rest of the refined twin fractions, mirror in (101) and inversion twin, returned meaningful values. The final refinement converged to *R* = 4.24% for 2344 unique observed reflections with *I* > 3σ(*I*) and GOF = 1.48. Statistical details for the refinement are given in Table 1 ▸. Final atom coordinates and displacement parameters for agrinierite are listed in Table 5 ▸, selected interatomic distances in Table 6 ▸ and a bond-valence analysis in Table 7 ▸. The bond-valence analysis was performed following the procedure by Brown (2002 ▸, 2009 ▸) using bond-valence parameters provided by Gagné & Hawthorne (2015 ▸).

**Figure 2:**
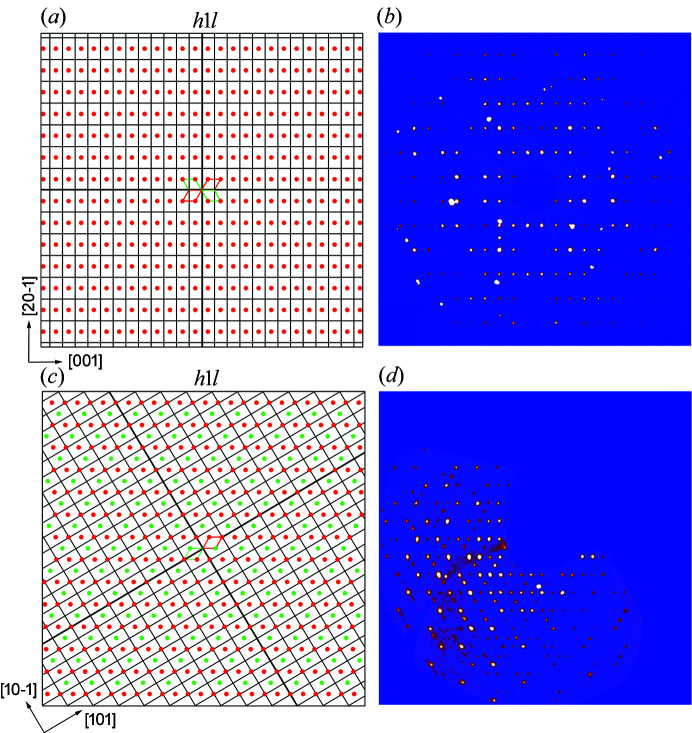
Single-crystal diffraction patterns of agrinierite and rameauite. (*a*) Simulated *h*1*l* layer of the reciprocal space of agrinierite. Reflections from all domains overlap completely (*i.e.* ‘hidden twinning’) due to twinning by metric merohedry and generate an *F*-centered (pseudo)orthorhombic pattern (the black array corresponds to the unit cell given by the previous structure determination). (*b*) Reciprocal space reconstruction (*UNWARP* tool) of the *h*1*l* layer from the experimental data for agrinierite. (*c*) Simulated *h*1*l* layer of the reciprocal space of rameauite. Reflections for the two main domains overlap only partially, due to twinning by reticular merohedry. The array corresponds to the supercell. (*d*) Reciprocal space reconstruction (*UNWARP* tool) of the *h*1*l* layer from the experimental data for rameauite.

Twin contributions for both minerals were evaluated also visually using the reciprocal layer reconstructions retrieved from the diffraction frames (the *UNWARP* tool within the *CrysAlis* software) and by computer methods using the program *Jana2020* (Figs. 2 ▸). We have chosen the best representatives for twinning in both minerals to be displayed. Figs. 2 ▸(*a*) and 2 ▸(*b*) display the *h*1*l* layer of the reciprocal space in agrinierite, with apparently all reflections overlapping. This makes the recognition of the twin presence relatively difficult, at least more difficult than in the case of rameauite [Figs. 2 ▸(*c*) and 2(*d*)]. Although the diffraction intensities are vastly affected by the twin contributions, at least some of the observed reflections that are diagnostic (*e.g.* Petříček *et al.*, 2016 ▸), *i.e.* warning us of twinning, are ‘visible’ (*i.e.* are not completely overlapping as in the case of agrinierite).

For the evaluation of the twin type studied here, it is both necessary and useful to transform the *C*-centered unit cells into primitive ones. Otherwise, the results of the test for the higher-symmetry cell in *Jana2020* will give correct results in terms of the searched cell, but the twin matrices will be applied to the conditions of the cell centering of our choice (and thus could be different from those without the applied conditions for centering and the systematic absences of reflections for the chosen space group). After *C*→*P* cell transformation the twin matrix of the mirror element for agrinierite is |1 0 0|0 1 0|1 0 1|, a mirror in (102), and for rameauite is |1/2 1/2 1/2|1/2 1/2 1/2|1 1 0|, a mirror in (1 11). Therefore, as the twin matrix for rameauite contains non-rational numbers, it appears to be twinned by reticular merohedry with apparent obliquity (diffraction type II). Agrinierite, with a matrix containing only rational numbers, thus appears to be twinned by metric merohedry [diffraction type I; see Petříček *et al.* (2016 ▸) for details]. To conclude, this is also the main reason for the distinct diffraction patterns of agrinierite [Figs. 2 ▸(*a*) and 2(*b*)] and rameauite [Figs. 2 ▸(*c*) and 2(*d*)]. In the case of agrinierite all reflections overlap (all displayed in red in the simulated pattern), whereas for agrinierite, they are separated (red and green) and form the pattern characteristic for this type of twin (we can call it an ‘hourglass’-like pattern; Petříček *et al.*, 2016 ▸).

### 3.2. The refined structures of agrinierite and rameauite

The current structure model of agrinierite leaves the findings of Cahill & Burns (2000 ▸) about structure topology unchanged. Nevertheless, as the correct structure crystallizes in the monoclinic *Cm* space group, the single *M* ^2+^-interlayer site in the model by Cahill & Burns (2000 ▸) is split into two symmetry non-equivalent sites. Moreover, Cahill & Burns (2000 ▸) restrained the occupation for Ca and Sr. The current model indicates that, while at one site (designated as *M*1) Sr is prevailing over Ca, at the second site (*M*2) Ca is slightly prevailing (Table 2 ▸). Therefore, we report the formula of agrinierite comprising two *M* ^2+^ sites as K_3.758_(Sr_0.89_Ca_0.11_)(Ca_0.57_Sr_0.43_)[(UO_2_)_3_O_3_(OH)_2_]_4_·10H_2_O, *Z* = 2. This formula is not electroneutral, having a 0.121 negative charge surplus; the scattering contribution of the K atoms, namely displacement parameters and occupation factors, is still probably vastly affected by twinning.

The same occurs for the structure model of rameauite, leaving the model proposed earlier by Plášil *et al.* (2016 ▸) unchanged in general. The fit to the data is better overall, as it can also be documented by the root-mean-squared deviation of the final bond-valence sums of the oxygen atoms within the structure (with the considered contribution of the *D*—H bonds, equal to 0.8 vu, for the H_2_O and OH groups equally for both rameauite structure models). For the structure model given by Plášil *et al.* (2016 ▸), this is 0.25 vu, and for the currently presented model it is 0.14 vu. The formula of rameauite, based on refined occupancies and bond-valence calculations, is K_4_Ca_2_[(UO_2_)_3_O_3_(OH)_2_]_4_·12H_2_O, *Z* = 2. We report the formula based on the same *Z* as for agrinierite to obtain a better comparison.

## 4. Implications: the careful handling of structures with ‘hidden’ twinning

The two uranyl-oxide hy­droxy-hydrate minerals presented here demonstrate how careful one must be when handling diffraction data affected by twinning. Agrinierite is representative of a structure providing a single-crystal diffraction pattern with hidden twinning. From the symmetry and diffraction intensities, it is difficult in this particular case to discover the twinning at first glance. Nevertheless, there are some general guides (*e.g.* Petříček *et al.*, 2016 ▸; Plášil *et al.*, 2021 ▸) that are still valid:

(1) Awkward cell centering (systematic absences generated by the twinning) for the given symmetry of the structure affected by (unresolved) twinning. In the case of agrinierite it was an *F*-centered orthorhombic cell.

(2) Higher residuals. In the case of agrinierite *R*_obs_ > 6% along with the overestimated fit (*S* value from *SHELX* < 1 for the given weighting scheme).

Nevertheless, the handling of the twinned structures might not be as straightforward as for the untwinned structures. The second example, rameauite, is an illustrative case. The twinning features present in the structure (as documented in this study) were simply overlooked by one of the authors (JP). We emphasize that the tolerance limits (for maximal deviations for cell lengths and angles) had to be increased during the test for reticular twinning in *Jana2020* for rameauite (up to 0.25 Å for cell lengths and 0.35° for angles). Then the procedure found the supercell of the higher symmetry unambiguously. We recommend doing so for cases of the worst diffraction data quality (and ‘worse’ fitted unit-cell metrics, which could bias the algorithm). However, the presence has to be then verified every time by a reasonable and meaningful structure refinement. For complicated unusual cases, when ordinary indexing programs (like the algorithms in *CrysAlis*) for unit-cell search fail, we recommend using the *Jana2020* built-in indexing feature, *GrIndex*. It is a powerful tool, not only for finding the unit cell even from biased data but also for various cell transformations and projections of data.

## Supplementary Material

Crystal structure: contains datablock(s) general, agrinierite, rameauite. DOI: 10.1107/S1600576721009663/vb5019sup1.cif

Structure factors: contains datablock(s) agrinierite. DOI: 10.1107/S1600576721009663/vb5019sup2.hkl

Structure factors: contains datablock(s) rameauite. DOI: 10.1107/S1600576721009663/vb5019sup3.hkl

CCDC references: 2110502, 2115477

## Figures and Tables

**Table 1:** Details for the data collection and refinement for agrinierite and rameauite

	Agrinierite	Rameauite
Structural formula (sum)	K_3.758_(Sr_1.32_Ca_0.68_)[(UO_2_)_3_O_3_(OH)_2_]_4_(H_2_O)_10_	K_4_Ca_2_[(UO_2_)_3_O_3_(OH)_2_]_4_(H_2_O)_12_
*a*, *b*, *c* (Å); β (°)	14.069 (3), 14.220 (3), 13.967 (3); 120.24 (12)	13.947 (3), 14.300 (3), 13.888 (3); 118.50 (3)
*V* (Å^3^)	2414.2 (12)	2434.3 (11)
Space group	*Cm*	*Cc*
*Z*	2	2
*D*_calc_ (g cm^−3^) for the above formula	5.500	5.442
Temperature (K)	296	296
Wavelength (Å)	Mo *K*α, 0.71073	Mo *K*α, 0.71073
Crystal dimensions (µm)	122 × 72 × 20	93 × 56 × 28
Limiting θ angles (°)	3.25–31.20	5.15–27.75
Limiting Miller indices	−19 ≤ *h* ≤ 20, −20 ≤ *k* ≤ 20, −19 ≤ *l* ≤ 19	−18 ≤ *h* ≤ 11, −18 ≤ *k* ≤ 18, −18 ≤ *l* ≤ 16
No. of measured reflections	24 870	7145
No. of unique reflections	7186	3280
No. of observed reflections (criterion)	6545 [*I*_obs_ > 3σ(*I*)]	2344 [*I*_obs_ > 3σ(*I*)]
Completeness, *R*_int_	0.93, 0.045	0.93, 0.045
Absorption correction (mm^–1^), *T*_min_/*T*_max_	42.05, 0.198/1	40.44, 0.417/1
*F*_000_	3343	3336
Parameters refined, restraints, constraints	206, 0, 17	196, 0, 2
*R*, *wR* (obs)	0.0354, 0.0931	0.0423, 0.0843
*R*, *wR* (all)	0.0389, 0.0980	0.0596, 0.0912
GOF obs/all	1.07/1.07	1.48/1.34
Δρ_min_, Δρ_max_ (e Å^–3^)	−4.24, +4.58 (0.6 Å to U1)	−7.11, +5.41 (0.5 Å to U3)
Weighting scheme, weights	σ, *w* = 1/[σ^2^(*I*) + 0.003363*I* ^2^]	σ, *w* = 1/[σ^2^(*I*) + 0.000485*I* ^2^]
Twin fractions 1, 2, 3, 4	0.45 (2)/0.067 (13)/0.455 (13)/0.033 (13)	0.74 (4)/0.215 (4)/0.05 (4)
Twin matrices /1, 2, 3/	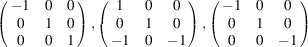	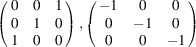

**Table 2:** Atom coordinates, isotropic and equivalent displacement parameters (Å), and site-occupancies for agrinierite

Atom	*x*/*a*	*y*/*b*	*z*/*c*	*U*_iso_*/*U*_eq_
U1	0.371711	0.759536	0.616595	0.0129(5)
U2	0.1928 (3)	0.73988 (5)	0.25716 (7)	0.013 (6)
U3	0.49507 (17)	0.7501 (2)	0.4369 (2)	0.0132 (5)
U4	0.37008 (12)	0.27269 (11)	0.11730 (11)	0.0122 (5)
U5	0.48845 (17)	0.23312 (3)	0.9369 (2)	0.0124 (4)
U6	0.6896 (2)	0.77272 (13)	0.75662 (13)	0.0156 (6)
*M*1†	0.3431 (4)	0	0.2494 (5)	0.028 (2)
*M*2†	0.5324 (5)	0.5	0.6242 (5)	0.013 (2)
K1	0.2984 (7)	0	0.9361 (14)	0.033 (4)
K2†	0.5259 (7)	1	0.5946 (7)	0.023 (2)*
K3	0.3674 (6)	0.5	0.2524 (7)	0.0318 (18)*
K4	0.8533 (10)	0	0.9345 (11)	0.076 (6)
O1	0.8224 (19)	0.7678 (7)	0.937 (3)	0.027 (2)*
O2	0.4620 (15)	0.1083 (7)	0.935 (3)	0.0238 (19)*
O3	0.3304 (12)	0.7403 (13)	0.4431 (18)	0.017 (3)*
O4	0.3569 (13)	0.1534 (12)	0.1235 (13)	0.021 (4)*
O5	0.5224 (17)	0.631 (2)	0.4820 (17)	0.026 (6)*
O6	0.4730 (17)	0.875 (2)	0.3874 (16)	0.021 (5)*
O7	0.3965 (11)	0.3964 (11)	0.1223 (12)	0.014 (3)*
O8	0.4087 (15)	0.6352 (17)	0.6341 (14)	0.011 (4)*
O9	0.0252 (12)	0.7539 (12)	0.1116 (12)	0.014 (3)*
O10	0.8480 (12)	0.7338 (11)	0.7641 (11)	0.012 (3)*
O11	0.1689 (14)	0.6167 (15)	0.2818 (14)	0.025 (4)*
O12	0.3426 (15)	0.8801 (15)	0.5998 (14)	0.024 (4)*
O13	0.6892 (14)	0.7804 (12)	0.5586 (14)	0.015 (4)*
O14	0.053 (2)	0.6939 (18)	0.8100 (19)	0.022 (6)*
O15	0.210 (2)	0.862 (2)	0.2340 (18)	0.030 (6)*
O16	0.3597 (11)	0.6927 (10)	0.2751 (10)	0.013 (3)*
O17	0.6544 (11)	0.6446 (12)	0.7537 (12)	0.016 (3)*
O18	0.5188 (16)	0.3568 (7)	0.935 (3)	0.0229 (19)*
O19	0.5684 (14)	0.7034 (13)	0.3192 (14)	0.020 (4)*
O20	0.1810 (16)	0.7015 (15)	0.0650 (16)	0.011 (5)*
O21	0.7125 (14)	0.8993 (14)	0.7710 (13)	0.029 (5)*
O22	0.5240 (10)	0.7855 (9)	0.6074 (10)	0.006 (2)*
O23	0.3292 (18)	0.5	0.4734 (19)	0.036 (6)*
O24	0.671 (2)	1	0.541 (2)	0.054 (8)*
O25	0.5839 (19)	0.5	0.3128 (19)	0.037 (6)*
O26	0.185 (2)	0	0.059 (2)	0.029 (7)*
O27	0.5427 (19)	0.5	0.8131 (19)	0.026 (6)*
O28	0.675 (2)	0	1.055 (2)	0.062 (8)*
O29	0.482 (2)	0	0.179 (2)	0.051 (7)*
O30	0.503 (2)	0	0.774 (2)	0.051 (7)*
O31	0.8303 (18)	0.5	0.4306 (17)	0.024 (4)*
O32	0.7210 (18)	0.5	0.6618 (19)	0.034 (6)*

† Refined occupancies: *M*1 0.89 (3)Sr/0.11 (3)Ca; *M*2 0.43 (3)Sr/0.57 (3)Ca; K2 0.76 (2).

**Table 3:** Selected interatomic distances (Å) in the structure of agrinierite

U1–O8	1.83 (3)	U2—O11	1.845 (15)	U3—O5	1.78 (3)
U1—O12	1.748 (14)	U2—O15	1.80 (3)	U3—O6	1.88 (3)
U1—O3	2.21 (3)	U2—O3	2.33 (2)	U3—O3	2.37 (2)
U1—O10^ii^	2.247 (16)	U2—O9	2.214 (11)	U3—O13	2.418 (12)
U1—O13^ii^	2.343 (14)	U2—O16	2.335 (16)	U3—O16	2.249 (11)
U1—O14^iii^	2.70 (2)	U2—O19^ii^	2.453 (17)	U3—O19	2.432 (17)
U1—O22	2.244 (16)	U2—O20	2.66 (3)	U3—O22	2.256 (15)
〈U1—O_Ur_〉	1.79	〈U2—O_Ur_〉	1.82	〈U3—O_Ur_〉	1.83
〈U1—O_eq_〉	2.35	〈U2—O_eq_〉	2.40	〈U3—O_eq_〉	2.35
					
U4—O4	1.720 (14)	U5—O2	1.811 (11)	U6—O17	1.879 (14)
U4—O7	1.785 (14)	U5—O18	1.814 (13)	U6—O21	1.819 (14)
U4—O1^vii^	2.26 (4)	U5—O1^ix^	2.39 (3)	U6—O1	2.26 (3)
U4—O9^viii^	2.233 (16)	U5—O9^x^	2.251 (15)	U6—O10	2.247 (16)
U4—O16^i^	2.329 (16)	U5—O10^ix^	2.220 (11)	U6—O13	2.758 (16)
U4—O19^i^	2.812 (2)	U5—O14^viii^	2.43 (3)	U6—O14^iii^	2.43 (3)
U4—O20^i^	2.41 (3)	U5—O20^x^	2.42 (2)	U6—O22	2.219 (10)
〈U4—O_Ur_〉	1.75	〈U5—O_Ur_〉	1.81	〈U6—O_Ur_〉	1.85
〈U4—O_eq_〉	2.35	〈U5—O_eq_〉	2.34	〈U6—O_eq_〉	2.38
					
*M*1—O4	2.861 (16)	*M*2—O5	2.67 (3)	K1—O2	2.78 (2)
*M*1—O4^xii^	2.861 (16)	*M*2—O5^i^	2.67 (3)	K1—O2^xii^	2.78 (2)
*M*1—O6^xiii^	2.58 (2)	*M*2—O8	2.64 (3)	K1—O4^xv^	3.182 (14)
*M*1—O6^i^	2.58 (2)	*M*2—O8^i^	2.64 (3)	K1—O4^xvi^	3.182 (14)
*M*1—O15^xiii^	2.65 (2)	*M*2—O17	2.710 (13)	K1—O17^x^	3.105 (14)
*M*1—O15^i^	2.65 (2)	*M*2—O17^i^	2.710 (13)	K1—O17^xiv^	3.105 (14)
*M*1—O26	2.46 (2)	*M*2—O23	2.56 (2)	K1—O26^xv^	2.87 (4)
*M*1—O29	2.59 (4)	*M*2—O27	2.57 (3)	K1—O27^x^	3.11 (3)
*M*1—O31^x^	2.63 (3)	*M*2—O32	2.43 (3)	K1—O29^xv^	3.06 (2)
〈*M*1—O〉	2.65	〈*M*2—O〉	2.62	K1—O32^x^	3.41 (3)
				〈K1—O〉	3.19
					
K2—O6	3.15 (2)	K3—O5	3.39 (2)	K4—O1^xiii^	3.334 (11)
K2—O6^xvii^	3.15 (2)	K3—O5^i^	3.39 (2)	K4—O1^i^	3.334 (11)
K2—O12	3.12 (3)	K3—O7	2.531 (18)	K4—O7^xi^	2.801 (19)
K2—O12^xvii^	3.12 (3)	K3—O7^i^	2.531 (18)	K4—O7^xix^	2.801 (19)
K2—O21	2.921 (13)	K3—O11	3.449 (18)	K4—O18^viii^	3.09 (2)
K2—O21^xvii^	2.921 (13)	K3—O11^i^	3.449 (18)	K4—O18^xx^	3.09 (2)
K2—O22	3.056 (13)	K3—O16	2.771 (14)	K4—O21^xiii^	2.571 (14)
K2—O22^xvii^	3.056 (13)	K3—O16^i^	2.771 (14)	K4—O21^i^	2.571 (14)
K2—O24	2.50 (4)	K3—O23	3.39 (3)	K4—O27^ix^	3.81 (4)
K2—O30^iv^	2.68 (3)	K3—O25	2.72 (3)	K4—O28	3.66 (10)
K2—O31^v^	2.54 (2)	K3—O28^vi^	2.72 (2)	〈K4—O〉	3.11
〈K2—O〉	2.93	〈K3—O〉	2.99		

Symmetry codes: (i) *x*, −*y* + 1, *z*; (ii) *x* − \textstyle {1\over2}, −*y* + \textstyle {3\over2}, *z*; (iii) *x* + \textstyle {1\over2}, −*y* + \textstyle {3\over2}, *z*; (iv) *x* − \textstyle {1\over2}, *y* + \textstyle {1\over2}, *z*; (v) *x* − \textstyle {1\over2}, *y* + \textstyle {1\over2}, *z* − 1; (vii) *x* − \textstyle {1\over2}, *y* − \textstyle {1\over2}, *z* − 1; (viii) *x* + \textstyle {1\over2}, *y* − \textstyle {1\over2}, *z*; (ix) *x* − \textstyle {1\over2}, *y* − \textstyle {1\over2}, *z*; (x) *x* + \textstyle {1\over2}, *y* − \textstyle {1\over2}, *z* + 1; (xi) *x*, −*y*, *z*; (xii) *x*, *y* − 1, *z*; (xiii) *x* − \textstyle {1\over2}, −*y* + \textstyle {1\over2}, *z*; (xiv) *x*, *y*, *z* + 1; (xv) *x*, −*y*, *z* + 1; (xvi) *x*, −*y* + 2, *z*; (xviii) *x*, *y* + 1, *z*; (xix) *x* + \textstyle {1\over2}, −*y* + \textstyle {1\over2}, *z* + 1; (xx) *x* + \textstyle {1\over2}, −*y* + \textstyle {1\over2}, *z*; (xxi) *x* + \textstyle {1\over2}, *y* + \textstyle {1\over2}, *z* + 1.

**Table 4:** Bond-valence analysis (all values given in valence units, vu) for agrinierite The bond-valence parameters were taken from Gagné & Hawthorne (2015 ▸). H – including a contribution of donor–hydrogen bonds; *n*H – maximum number of possible weak H⋯acceptor bonds to a particular site. Idealized bond strengths were taken from Brown (2002 ▸). Donor–H (0.8 vu), H⋯acceptor (0.2 vu). Site occupancies considered.

	U1	U2	U3	U4	U5	U6	*M*1	*M*2	K1	K2	K3	K4	Sum	Sum^H^	*n*H
O1				0.64	0.48	0.64			0.02			0.08	1.86	1.86	1
O2					1.64				0.32				1.96	1.96	0
O3	0.71	0.55	0.50										1.76	1.76	1
O4				1.99			0.29		0.12				2.20	2.20	0
O5			1.75					0.37			0.07		1.93	1.93	0
O6			1.42				0.53			0.10			1.72	1.72	1
O7				1.74							0.59	0.30	2.18	2.18	0
O8	1.58							0.40					1.78	1.78	1
O9		0.70		0.67	0.65								2.02	2.02	0
O10	0.65				0.69	0.65							2.00	2.00	0
O11		1.53									0.06		1.59	1.59	2
O12	1.88									0.10			1.93	1.93	0
O13	0.53		0.46			0.22							1.21	2.01	0
O14	0.25				0.44	0.44							1.14	1.94	0
O15		1.68					0.46						1.91	1.91	0
O16		0.54	0.65	0.55							0.32		1.91	1.91	0
O17						1.42		0.34	0.14				1.66	1.66	2
O18					1.63							0.15	1.71	1.71	1
O19		0.42	0.44	0.20									1.06	1.86	1
O20		0.27		0.46	0.45								1.19	1.99	0
O21						1.62				0.17		0.54	1.97	1.97	0
O22	0.66		0.64			0.69				0.12			2.05	2.05	0
O23								0.24			0.03		0.27	1.87	1
O24										0.24			0.24	2.04	0
O25											0.18		0.18	1.78	1
O26							0.34		0.13				0.47	2.07	0
O27								0.23	0.07			0.01	0.31	1.91	0
O28											0.18	0.02	0.20	1.80	1
O29							0.26		0.08				0.34	1.94	0
O30										0.15			0.15	1.75	1
O31							0.24			0.22			0.46	2.06	0
O32								0.32	0.03				0.35	1.95	0
Sum	6.26	5.70	5.87	6.25	6.00	5.69	2.11	1.90	0.90	1.11	1.45	1.09			

**Table 5:** Atom coordinates and isotropic and equivalent displacement parameters (Å) for rameauite

Atom	*x*/*a*	*y*/*b*	*z*/*c*	*U*_iso_*/*U*_eq_
U1	0.2214 (11)	0.50151 (9)	0.1010 (10)	0.0168 (6)
U2	0.0881 (11)	0.52239 (9)	0.2828 (10)	0.0196 (6)
U3	0.2102 (11)	0.00417 (9)	0.0935 (10)	0.0173 (5)
U4	0.4099 (11)	0.51689 (10)	0.4037 (10)	0.0173 (5)
U5	0.0692 (11)	−0.01326 (10)	0.2565 (10)	0.0170 (6)
U6	0.9025 (11)	0.51586 (10)	0.9074 (10)	0.0185 (6)
Ca1	0.0961 (13)	0.2526 (6)	0.1526 (12)	0.031 (3)
K1	0.2419 (13)	0.7507 (6)	0.2578 (12)	0.035 (3)
O1	0.200 (2)	0.3786 (16)	0.123 (2)	0.027 (6)*
O2	0.076 (2)	0.5070 (19)	0.429 (2)	0.026 (6)*
O3	0.092 (2)	0.6491 (13)	0.297 (2)	0.015 (4)*
K2	0.0953 (14)	0.7511 (7)	0.9147 (13)	0.037 (4)
O4	0.395 (2)	0.4876 (16)	0.2248 (19)	0.023 (6)*
O5	0.579 (2)	0.5040 (17)	0.9191 (19)	0.019 (5)*
O6	0.539 (2)	0.5270 (17)	0.584 (2)	0.018 (5)*
O7	0.038 (2)	0.5519 (17)	0.0945 (19)	0.024 (5)*
O8	0.238 (2)	0.5312 (18)	0.266 (2)	0.026 (6)*
O9	0.899 (2)	0.5389 (17)	0.236 (2)	0.024 (6)*
O10	0.028 (2)	0.747 (2)	0.084 (2)	0.033 (7)*
O11	0.283 (2)	0.5487 (17)	0.4747 (19)	0.031 (5)*
O12	0.240 (2)	0.623 (2)	0.082 (2)	0.037 (7)*
O13	0.892 (3)	0.396 (2)	0.942 (3)	0.052 (9)*
O14	0.419 (2)	0.8612 (14)	0.3798 (19)	0.015 (5)*
O15	0.735 (2)	0.518 (2)	0.763 (2)	0.038 (7)*
O16	0.081 (2)	0.3996 (17)	0.258 (2)	0.029 (7)*
O17	0.191 (2)	0.1289 (15)	0.110 (2)	0.022 (5)*
O18	0.073 (2)	0.1140 (14)	0.250 (2)	0.018 (5)*
O19	0.393 (3)	0.3907 (17)	0.420 (2)	0.031 (6)*
O20	0.253 (3)	0.2505 (17)	0.334 (3)	0.033 (6)*
O21	0.049 (3)	0.2527 (18)	−0.039 (2)	0.041 (8)*
O22	−0.111 (3)	0.7486 (18)	0.217 (3)	0.035 (7)*
O23	0.244 (2)	−0.1166 (19)	0.087 (2)	0.020 (6)*
O24	0.422 (2)	0.6448 (16)	0.391 (2)	0.031 (6)*
O25	0.073 (2)	−0.1438 (15)	0.2669 (18)	0.024 (6)*
O26	0.795 (2)	0.5542 (16)	0.9874 (19)	0.028 (5)*
O27	−0.231 (2)	0.7514 (19)	0.018 (2)	0.035 (7)*
O28	−0.086 (3)	0.254 (2)	0.127 (3)	0.055 (8)*

**Table 6:** Selected interatomic distances (Å) in the structure of rameauite

U1—O1	1.83 (3)	U2—O3	1.819 (15)	U3—O17	1.83 (3)
U1—O12	1.80 (3)	U2—O16	1.78 (3)	U3—O23	1.81 (3)
U1—O2^i^	2.28 (2)	U2—O7	2.41 (3)	U3—O5^vii^	2.24 (2)
U1—O4	2.21 (2)	U2—O8	2.22 (4)	U3—O6^vi^	2.36 (3)
U1—O7	2.61 (3)	U2—O9^iv^	2.41 (3)	U3—O9^v^	2.47 (2)
U1—O8	2.23 (3)	U2—O11	2.78 (2)	U3—O15^vi^	2.22 (3)
U1—O11^i^	2.40 (4)	〈U2—O_Ur_〉	1.80	U3—O26^vii^	2.41 (4)
〈U1—O_Ur_〉	1.81	〈U2—O_eq_〉	2.39	〈U3—O_Ur_〉	1.82
〈U1—O_eq_〉	2.35			〈U3—O_eq_〉	2.34
					
U4—O19	1.84 (3)	U5—O18	1.825 (15)	U6—O13	1.81 (3)
U4—O24	1.86 (3)	U5—O25	1.87 (3)	U6—O14^ix^	1.839 (18)
U4—O4	2.43 (3)	U5—O4^v^	2.25 (3)	U6—O2^x^	2.32 (3)
U4—O5^i^	2.28 (3)	U5—O5^vi^	2.19 (3)	U6—O7^xi^	2.43 (2)
U4—O6	2.28 (2)	U5—O6^vi^	2.24 (3)	U6—O9^xii^	2.48 (3)
U4—O8	2.25 (2)	U5—O15^vi^	2.28 (3)	U6—O15	2.23 (2)
U4—O11	2.45 (4)	〈U5—O_Ur_〉	1.85	U6—O26	2.31 (4)
〈U4—O_Ur_〉	1.85	〈U5—O_eq_〉	2.24	〈U6—O_Ur_〉	1.83
〈U4—O_eq_〉	2.33			〈U6—O_eq_〉	2.36
					
Ca1—O1	2.47 (3)	K1—O3	2.80 (3)	K2—O5^xvi^	3.63 (3)
Ca1—O13^ii^	3.60 (3)	K1—O8	3.15 (3)	K2—O10^xvii^	2.91 (4)
Ca1—O16	2.63 (3)	K1—O10	2.81 (2)	K2—O12^xvii^	2.89 (3)
Ca1—O17	2.45 (3)	K1—O12	3.04 (3)	K2—O14^xviii^	2.77 (3)
Ca1—O18	2.50 (3)	K1—O14	2.73 (2)	K2—O16^xii^	3.01 (3)
Ca—O20	2.42 (3)	K1—O15^xiv^	3.31 (3)	K2—O18^xii^	2.90 (3)
Ca1—O21	2.42 (3)	K1—O23^xv^	3.05 (3)	K2—O20^xii^	2.91 (5)
Ca1—O28	2.39 (5)	K1—O24	2.75 (3)	K2—O23^xix^	2.97 (3)
〈Ca1—O〉	2.61	K1—O25^xv^	2.85 (3)	K2—O24^xviii^	2.72 (3)
		K1—O27^ix^	2.95 (3)	K2—O28^xii^	3.57 (4)
		〈K1—O〉	2.97	K1—O33^ix^	3.46 (3)
				〈K1—O〉	3.03

Symmetry codes: (i) *x*, −*y* + 1, *z* − \textstyle {1\over2}; (ii) *x* − 1, *y*, *z* − 1; (iv) *x* − 1, *y*, *z*; (v) *x* − \textstyle {1\over2}, *y* − \textstyle {1\over2}, *z*; (vi) *x* − \textstyle {1\over2}, −*y* + \textstyle {1\over2}, *z* − \textstyle {1\over2}; (vii) *x* − \textstyle {1\over2}, *y* − \textstyle {1\over2}, *z* − 1; (ix) *x* + \textstyle {1\over2}, −*y* + \textstyle {3\over2}, *z* + \textstyle {1\over2}; (x) *x* + 1, −*y* + 1, *z* + \textstyle {1\over2}; (xi) *x* + 1, *y*, *z* + 1; (xii) *x*, −*y* + 1, *z* + \textstyle {1\over2}; (xiv) *x* − \textstyle {1\over2}, −*y* + \textstyle {3\over2}, *z* − \textstyle {1\over2}; (xv) *x*, *y* + 1, *z*; (xvi) *x* − \textstyle {1\over2}, *y* + \textstyle {1\over2}, *z*; (xvii) *x*, *y*, *z* + 1; (xviii) *x* − \textstyle {1\over2}, −*y* + \textstyle {3\over2}, *z* + \textstyle {1\over2}; (xix) *x*, *y* + 1, *z* + 1.

**Table 7:** Bond-valence analysis (all values given in vu) for rameauite The bond-valence parameters were taken from Gagné & Hawthorne (2015 ▸). H – including a contribution of donor–hydrogen bonds; *n*H – maximum number of possible weak H⋯acceptor bonds to the particular site. Idealized bond strengths were taken from Brown (2002 ▸) – donor–H (0.8 vu), H⋯acceptor (0.2 vu).

	U1	U2	U3	U4	U5	U6	Ca1	K1	K2	Sum	Sum^H^	*n*H
O1	1.61						0.25			1.86	1.86	1
O2	0.61	0.86				0.56				2.03	2.03	0
O3		1.62						0.15		1.77	1.77	1
O4	0.71			0.44	0.65					1.80	1.80	1
O5			0.66	0.61	0.74				0.02	2.03	2.03	0
O6			0.51	0.61	0.66					1.79	1.79	1
O7	0.30	0.47				0.43				1.21	2.01	0
O8	0.68	0.69		0.65				0.06		2.08	2.08	0
O9		0.46	0.41			0.40				1.27	2.07	0
O10									0.11	0.11	1.71	1
O11	0.46	0.21		0.44						1.11	1.91	0
O12	1.68							0.15	0.12	1.95	1.95	0
O13						1.65	0.02			1.66	1.66	2
O14						1.55		0.08	0.16	1.79	1.79	1
O15			0.69		0.61	0.68		0.18		2.16	2.16	0
O16		1.75					0.17		0.09	2.01	2.01	0
O17			1.58				0.27			1.84	1.84	1
O18					1.60		0.23		0.12	1.95	1.95	0
O19				1.55						1.55	1.55	2
O20							0.29		0.11	0.40	2.00	0
O21							0.29			0.29	1.89	1
O22										0.00	1.60	2
O23			1.65					0.04	0.10	1.79	1.79	1
O24				1.48				0.08	0.18	1.75	1.75	1
O25					1.44			0.13		1.57	1.57	2
O26			0.46			0.57				1.04	1.84	1
O27								0.10		0.10	1.70	1
O28							0.31		0.02	0.33	1.93	0
Sum	6.05	6.06	5.97	5.78	5.70	5.84	1.82	0.98	1.04			
